# Variations of partial anomalous pulmonary venous connection

**DOI:** 10.1093/ehjcr/ytaf133

**Published:** 2025-03-25

**Authors:** Leqing Chen, Yukun Cao, Lixia Wang, Heshui Shi

**Affiliations:** Department of Radiology, Union Hospital, Tongji Medical College, Huazhong University of Science and Technology, 1277 Jiefang Rd, Wuhan 430022, China; Hubei Province Key Laboratory of Molecular Imaging, Union Hospital, Tongji Medical College, Huazhong University of Science and Technology, 1277 Jiefang Rd, Wuhan 430022, China; Department of Radiology, Union Hospital, Tongji Medical College, Huazhong University of Science and Technology, 1277 Jiefang Rd, Wuhan 430022, China; Hubei Province Key Laboratory of Molecular Imaging, Union Hospital, Tongji Medical College, Huazhong University of Science and Technology, 1277 Jiefang Rd, Wuhan 430022, China; Department of Radiology, Union Hospital, Tongji Medical College, Huazhong University of Science and Technology, 1277 Jiefang Rd, Wuhan 430022, China; Hubei Province Key Laboratory of Molecular Imaging, Union Hospital, Tongji Medical College, Huazhong University of Science and Technology, 1277 Jiefang Rd, Wuhan 430022, China; Department of Radiology, Union Hospital, Tongji Medical College, Huazhong University of Science and Technology, 1277 Jiefang Rd, Wuhan 430022, China; Hubei Province Key Laboratory of Molecular Imaging, Union Hospital, Tongji Medical College, Huazhong University of Science and Technology, 1277 Jiefang Rd, Wuhan 430022, China

Patient 1, a 50-year-old male with intermittent shortness of breath for 2 years (*Figure 1A*). CT pulmonary angiography revealed an anomalous connection between the right superior pulmonary vein (PV) and the superior vena cava (SVC), accompanied by mild pulmonary arterial hypertension (PAH) (Supplementary material online, *Figure S1*). The patient was managed conservatively and scheduled for regular follow-up examinations. Patient 2, a 59-year-old male, presented to our institution with bilateral lower limb oedema (*Figure 1B*). CT pulmonary angiography exhibited drainage of the right superior and middle PV into the SVC, along with PAH. Patient 3, a 40-year-old female, presented to our hospital complaining of chest tightness after exercise (*Figure 1C*). Transthoracic echocardiography revealed an enlarged right cardiac chamber and moderate tricuspid regurgitation. Subsequent CTPA demonstrated the left superior and lower PV draining into the left innominate vein, and the right superior and middle PV draining into the SVC. An atrial septal defect (ASD) was also detected, accompanied by severe PAH. This patient successfully underwent surgical correction.

**Figure 1:**
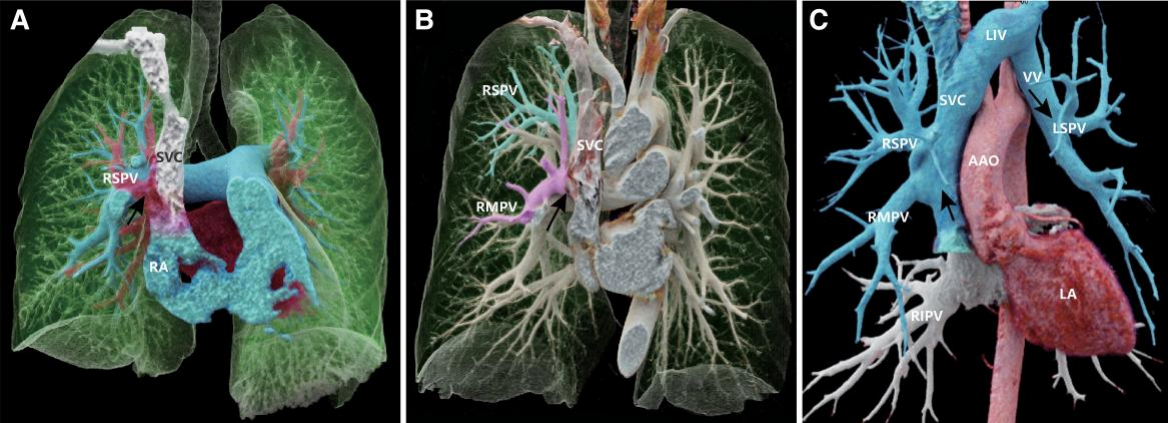
The cinematic rendering views show anomalous pulmonary venous connections. *(A)* (Patient 1): Anomalous connection between right superior pulmonary vein and superior vena cava; *(B)* (Patient 2): Drainage of right superior and middle pulmonary vein into superior vena cava; *(C)* (Patient 3): Left superior and lower pulmonary vein to left innominate vein, right superior, and middle pulmonary vein to superior vena cava. AAO, ascending aorta; LIV, left innominate vein; LSPV, the left superior pulmonary vein; RA, right atrium; RIPV, right inferior pulmonary vein; RMPV, right middle pulmonary vein; RSPV, right superior pulmonary vein; SVC, superior vena cava; VV, vertical vein.

Isolated PAPVC without ASD (Patient 1) as well as bilateral anomalous connection (Patient 3) are extremely rare anatomical findings. Partial anomalous pulmonary venous connection may develop symptoms such as PAH or right-sided volume overload depending on the number of abnormal connections.^1^ Compared to other existing studies, we visually present the relationship between different degrees of PAPVC and PAH.^2^ Although MRI enables Qp:Qs calculation and doesn't require iodine contrast administration, CTPA offers detailed anatomical information with high spatial resolution quickly and having broader availability. In cases of PAPVC without significant associated complications, regular follow-up with echocardiography to monitor pulmonary pressure and heart function is essential (Patients 1 and 2). For more severe cases involving ASD or severe PAH (Patient 3), surgical intervention may be considered to correct the anomalous connections and repair the septal defect.^3^

## Supplementary Material

ytaf133_Supplementary_Data

## Data Availability

Data will be made available on request.

## Lead author biography

Leqing Chen is currently pursuing a PhD degree in the Department of Radiology at Union Hospital, Tongji Medical College, Huazhong University of Science and Technology. The main research focuses on thoracic imaging and cardiovascular imaging.

## Supplementary material

**Consent:** The authors confirm that written consent for submission and publication of this case report including images and associated text has been obtained from the patient in line with COPE guidance.

**Funding:** This study was supported by the Clinic Research Fund of Union Hospital (grant number: 2021xhlcj08).
