# Supplementary material for: Variations of partial anomalous pulmonary venous connection
Source: Eur Heart J Case Rep. 2025 Mar 25;9(4):ytaf133. doi: 10.1093/ehjcr/ytaf133 (PMC11969213; doi:10.1093/ehjcr/ytaf133)
Supplement: ytaf133_Supplementary_Data [file ytaf133_supplementary_data.docx]

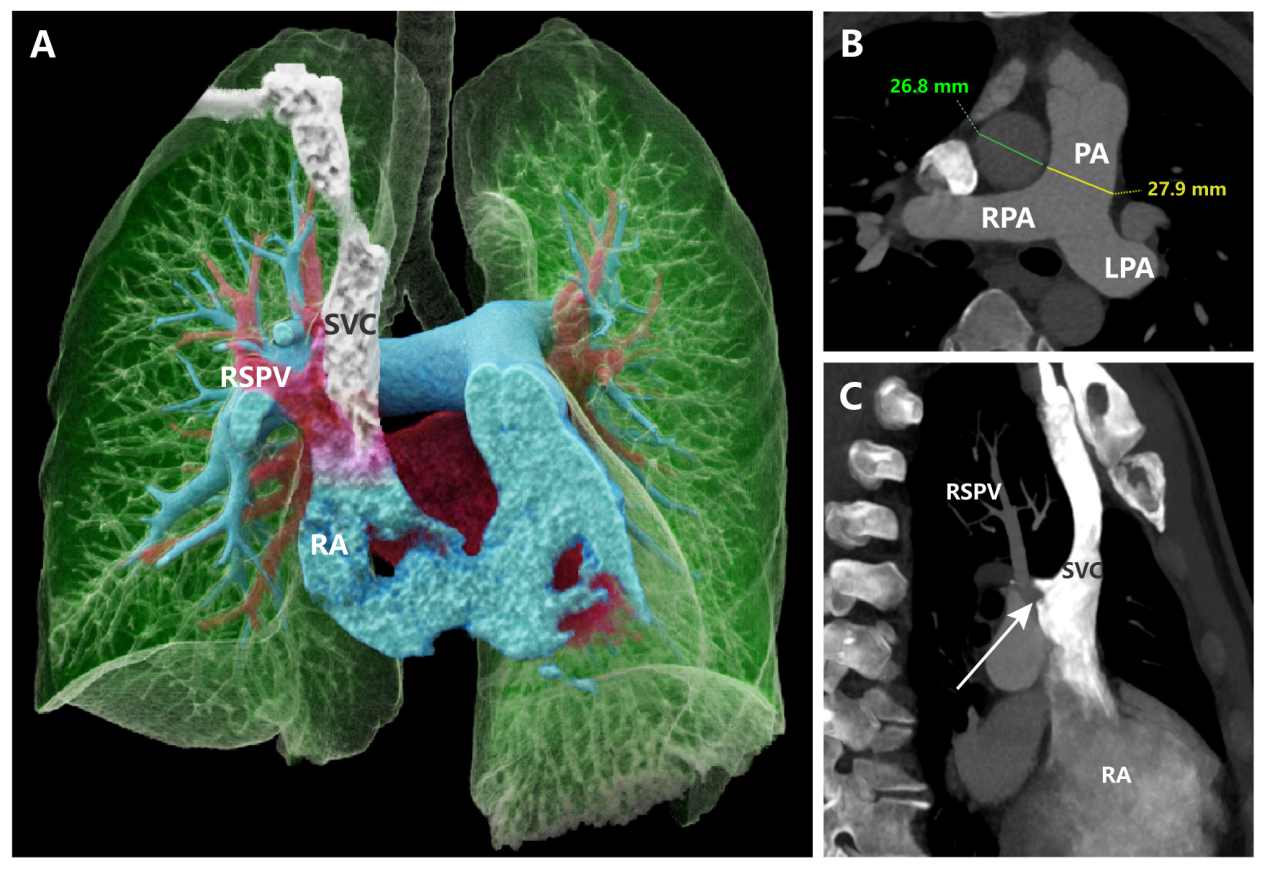


Figure S1

A: The cinematic rendering view illustrated abnormal drainage of the pulmonary vein in the right superior pulmonary vein to the superior vena cava.

B: The pulmonary artery was slightly widened (PAD, 27.9mm; PA:A ratio, 1.04).

C: The maximum intensity projection (MIP) images clearly display the inlet of the pulmonary vein with abnormal drainage (arrow).

Abbreviation: LPA: left pulmonary artery; PA, pulmonary artery; PAD, pulmonary artery diameter; PA:A ratio, pulmonary artery to aorta ratio; RA, right atrium; RPA: right pulmonary artery; RSPV, right superior pulmonary vein; SVC, superior vena cava.


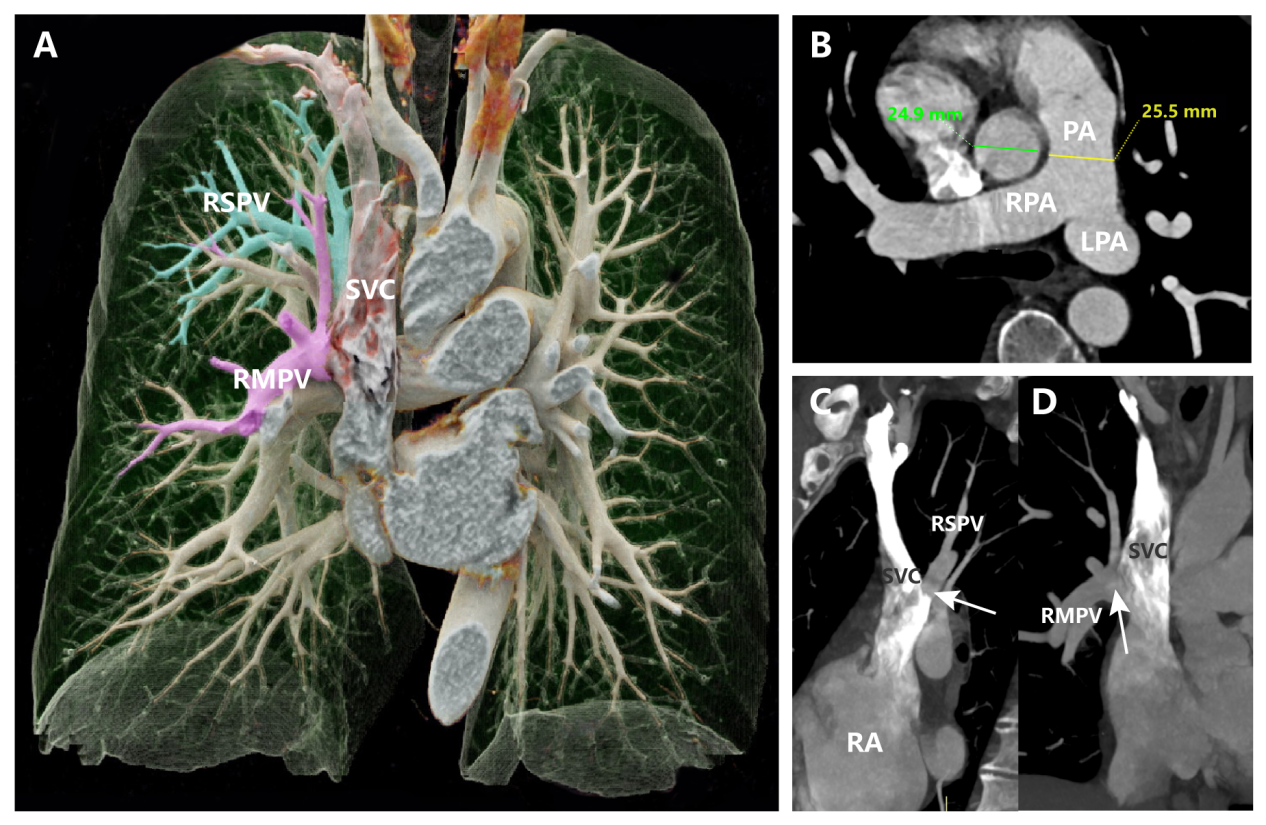


Figure S2

A: The cinematic rendering view showed abnormal venous drainage in right superior lobe (blue) and middle lobe (pink) pulmonary vein

B: The pulmonary artery was slightly widened (PAD, 25.5mm; PA:A ratio, 1.02).

C and D: The maximum intensity projection (MIP) images clearly display the inlet of the pulmonary vein with abnormal drainage (arrow).

Abbreviation: LPA: left pulmonary artery; PA, pulmonary artery; PAD, pulmonary artery diameter; PA:A ratio, pulmonary artery to aorta ratio; RA, right atrium; RMPV, right middle pulmonary vein; RPA: right pulmonary artery; RSPV, right superior pulmonary vein; SVC, superior vena cava.


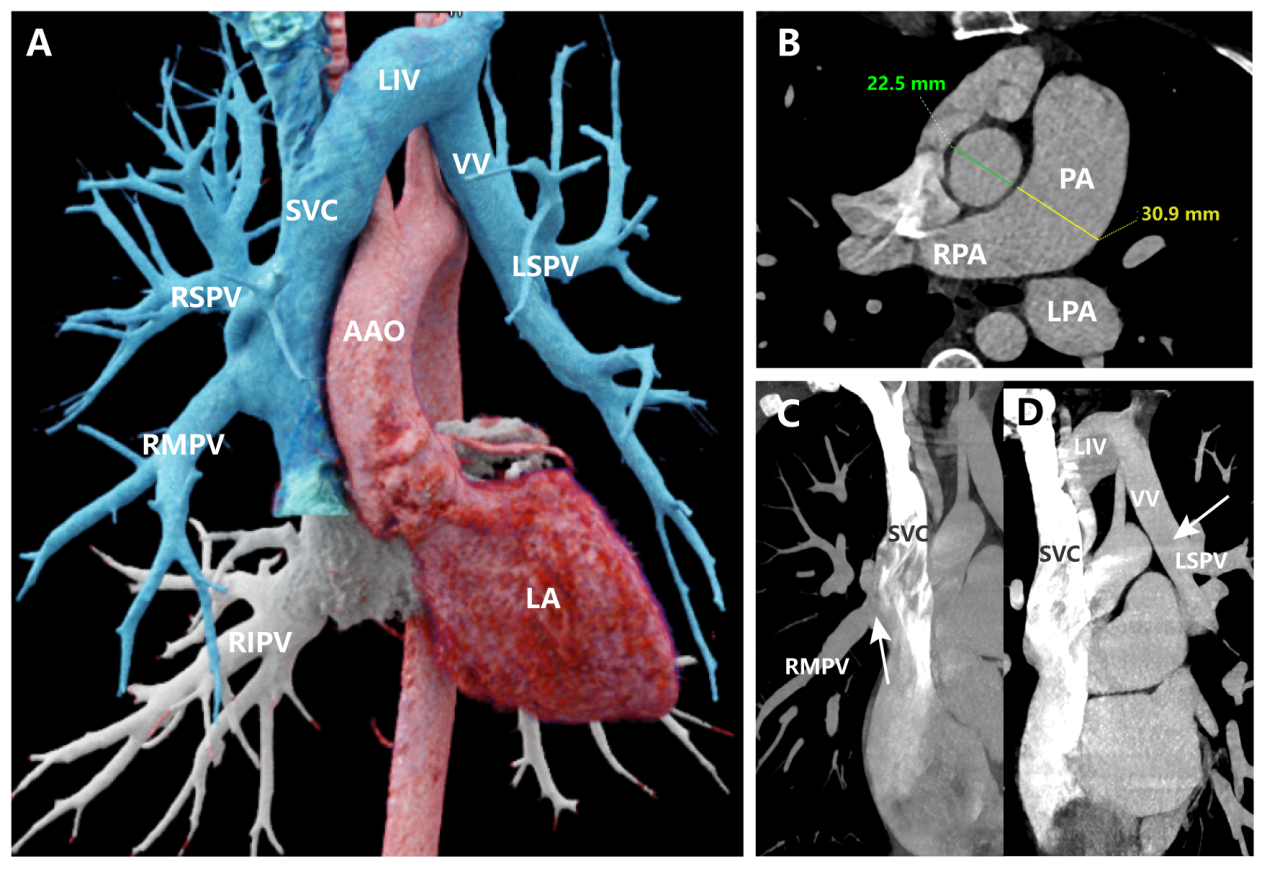


Figure S3

A: The cinematic rendering view illustrated the left superior and lower pulmonary veins draining into the left innominate vein, and the right superior and middle pulmonary veins draining into the SVC.

B: The pulmonary artery was significantly widened (PAD, 30.9mm; PA:A ratio, 1.37).

C and D: The maximum intensity projection (MIP) images clearly display the inlet of the pulmonary vein with abnormal drainage (arrow).

Abbreviation: AAO, ascending aorta; LIV, left innominate vein; LPA: left pulmonary artery; LSPV, the left superior pulmonary vein; PA, pulmonary artery; PAD, pulmonary artery diameter; RA, right atrium; RIPV, right inferior pulmonary vein; RMPV, right middle pulmonary vein; RPA: right pulmonary artery; RSPV, right superior pulmonary vein; SVC, superior vena cava; VV, vertical vein.
